# Analysis of the Readability of Questionnaires on Symptoms of Pelvic Floor Dysfunctions Adapted to Spanish

**DOI:** 10.3390/ijerph181910320

**Published:** 2021-09-30

**Authors:** Víctor Checa-Moreno, Esther Díaz-Mohedo, Carmen Suárez-Serrano

**Affiliations:** 1 Essere Vida y Salud, Clínica de Fisioterapia, Osteopatía y Psicología, 18009 Granada, Spain; fisioterapiavictorcheca@gmail.com; 2 Department of Physiotherapy, University of Málaga, 29071 Málaga, Spain; 3 Department of Physiotherapy, University of Seville, 41009 Seville, Spain; csuarez@us.es

**Keywords:** questionnaire, pelvic floor dysfunctions, urinary incontinence, fecal incontinence, overactive bladder, benign prostatic hyperplasia

## Abstract

Questionnaires are tools of interest in the evaluation of pelvic floor dysfunctions, but their success depends on their readability. Evaluating symptoms associated with such dysfunctions through questionnaires validated in Spanish with adequate readability indices will be useful for subsequent therapeutic work with these patients. The objective of this study is to evaluate the readability of symptomatology questionnaires on pelvic floor dysfunctions adapted to Spanish. This descriptive study included a total of 19 questionnaires, whose readability was analyzed according to four indices: Fernández-Huerta, Szigriszt-Pazos, Inflesz and readability µ (mu). In total, 50% of the questionnaires for fecal incontinence symptoms were found to have inadequate scores in terms of readability, according to the Inflesz index. If we take the readability mu index as a reference, the number of questionnaires that do not meet the minimum readability limit are as follows: 20% in urinary incontinence, 50% in fecal incontinence, 66.6% in sexual function, 100% in general pelvic floor, 25% in overactive bladder and 100% in benign prostatic hyperplasia. Therefore, it is necessary to review and adapt health questionnaires on pelvic floor dysfunctions to improve their readability and ease of understanding by conducting studies with the people who fill out these questionnaires.

## 1. Introduction

Up to a third of adult women may be affected by one of the three most common pelvic floor dysfunctions (urinary and fecal incontinence and prolapse of any of the pelvic organs) or a combination thereof [1]. Men may also be affected by pelvic floor dysfunctions, especially following prostate cancer surgery [2].

These dysfunctions most commonly present in the form of symptoms of gynecological origin (in women), of urinary and intestinal origin, and those that affect sexual function [3].

The diagnosis of pelvic floor dysfunctions must be precise and medical history and physical examination play an essential role in this. Obtaining objective data is essential in clinical history and health questionnaires should be collected, as they are useful tools in the detection of these health problems and in the determination of their impact [4].

The information that a person provides in a health questionnaire is beneficial to quantify symptoms, both in clinical practice and in research [5]. There are multiple validated and published questionnaires for different dysfunctions, both for purely technical aspects (to protocolize diagnoses, treatments, etc.) and for the determination of the impact on quality of life [6]. One of the most important issues to consider when it comes to health questionnaires is the length of the questions, which should, ideally, be as short as possible, since long questions can be perceived as tedious, take more time and distract participants [7]. Assessing the readability of questionnaires can also play an integral role in improving health, assisting in clinical decision making and providing accurate measures and results for treatment.

As regards this latter aspect, i.e., readability, authors such as Alas et al. [8] and Gaines and Malik [5] have shown that symptom questionnaires focused on the different dysfunctions of the pelvic floor in their original language, English, present certain deficiencies in their ability to be understood by affected patients, which would hinder their correct reading and, therefore, the answers provided.

Given that questionnaires translated from those developed in English require a cultural adaptation to the translated language, it is also necessary to know how this adaptation affects their readability.

Therefore, the objective of this study is to analyze the readability of the questionnaires and scales aimed at evaluating different pelvic floor dysfunctions and their associated symptoms in questionnaires adapted into and validated in Spanish.

## 2. Materials and Methods

This study analyzed the readability of symptom questionnaires on pelvic floor dysfunctions that have been adapted to Spanish.

### 2.1. Sample Selection

The questionnaires to be evaluated in this study were collected through a search carried out for instruments and questionnaires that evaluate the symptoms present in different pelvic floor dysfunctions. The following terms were used in this online search: urinary incontinence (UI), fecal incontinence (FI), pelvic organ prolapse (POP), sexual function (SF), overactive bladder (OAB), benign prostatic hyperplasia (BPH) and questionnaire.

The search was carried out by two independent investigators and, after the search, the authors conducted a review to verify that all the questionnaires included were appropriate and that no questionnaires had been missed.

All of the questionnaires were adjusted based on the following selection criteria.

#### 2.1.1. Inclusion Criteria

- Questionnaires that evaluate the symptoms of urinary incontinence, fecal incontinence, overactive bladder and benign prostate hyperplasia, as well as global questionnaires of pelvic floor dysfunctions;
- Questionnaires adapted to and validated in Spanish.

#### 2.1.2. Exclusion Criteria

- Questionnaires developed exclusively in Spanish;
- Questionnaires aimed at other pathologies that do not belong to the sphere of pelvic floor dysfunctions and that include those symptoms.

### 2.2. Selected Questionnaires

A total of 19 questionnaires were included that assess symptoms in the following dysfunctions:

- Five questionnaires for urinary incontinence: ICIQ-SF [9], UDI-6 [10], 3IQ [11], QUID [11] and Sandvik severity test [12];
- Four questionnaires for fecal incontinence: Wexner [13], Browning and Parks [13], FISI [13] and St Marks Hospital [14];
- Three questionnaires for sexual function in pelvic floor pathologies: FSFI [15], PISQ-IR [16] and PISQ-12 [17];
- Four questionnaires for overactive bladder: OABSS [18], SAQ [19], OAB V8 [20] and OAB V3 [20];
- A questionnaire for benign prostatic hyperplasia: IPSS [21];
- Two global symptom questionnaires on pelvic floor dysfunctions: EPIQ [22] and PFDI-20 [23].

Legibility is the variable that determines the ease of reading a questionnaire and that is used in the comparison between the different indices within the same questionnaire, as well as in the comparison between questionnaires that attempt to analyze the same dysfunction. With all this, it is possible to offer a clarifying vision regarding the questionnaires used for the evaluation and diagnosis of the different pelvic floor dysfunctions analyzed.

### 2.3. Readability Indexes

There were four different indices that we used to evaluate the readability of the questionnaires, explained below.

#### 2.3.1. Readability of Fernández Huerta

Readability, according to the author [24], is the linguistic readability of a text, that is, whether it is easy or difficult to understand. It does not cover typographical aspects that greatly influence the ease of reading. José Fernández Huerta created the second formula to measure the readability of texts in the Spanish language in 1959. This formula is based on that of Flesch (for English). The equation is

L = 206.84 − 0.60P − 1.02F

where L is the readability, P is the average number of syllables per word and F is the mean number of words per sentence [24].

It is necessary to indicate that, despite its extensive use in various articles and websites, this scale is not exempt from criticism, because Fernández Huerta did not explain the validation procedure or the procedure of validating the scale of interpretation of scores.

#### 2.3.2. The Szigriszt-Pazos Perspicuity Index

In 1993, journalist Francisco Szigriszt-Pazos [25] proposed a formula to measure readability in his doctoral thesis. This formula is an adaptation of the Flesch equation designed for English into Spanish. It is calculated as follows:

P = 206.835 − 62.3S/P − P/F

where P is the perspicuity, S refers to the total number of syllables, P refers to the number of words and F refers to the number of sentences. Perspicuity was defined as the quality of style that is intelligible, i.e., that can be understood.

#### 2.3.3. The Inflesz Readability Scale

This scale measures the ease of reading a text [26] and was developed by Inés María Barrio Cantalejo. It has been adapted to the current average Spanish reader and is calculated as perspicuity (Szigriszt-Pazos):

I = 206.835 − 62.3S/P − P/F

where I is the Inflesz scale, S refers to the total number of syllables, P refers to the number of words and F refers to the number of sentences [26].

The Inflesz scale has been used in the healthcare setting to assess the readability of informed consent, leaflets and health education materials. This legibility in written texts that are addressed to patients is an indicator of quality of care. The process of analysis of the legibility in these texts is linked to the progressive development of the idea of moral autonomy of patients to make decisions, the key concept of which is informed consent. Therefore, in order to create a new model of a clinical relationship based on the protagonist role of the patients themselves, research is key. Texts related to health have a greater probability of being read and understood, according to this scale, if they exceed a score of 55. Ultimately, materials written in this way fulfill the objective for which they were designed, i.e., to transmit the necessary information to the patient, allowing them to participate in making decisions that affect their health [27].

However, this scale can be applied to any text, even if it is not related to health.

#### 2.3.4. Readability µ (mu)

This is a formula that calculates the ease of reading a text, developed by Miguel Muñoz Baquedano and José Muñoz Urra in Chile in 2006 [28]. They included in the calculations the number of words and the mean and variance of the number of letters of said words.

The formula for readability µ (mu) is

$$\mu =\left(\frac{n}{n-1}\right)\left(\frac{\;\bar{x}}{\sigma^{2}}\right)$$

where *µ* is the readability index, *n* refers to the number of words, $\bar{x}$ is the mean number of letters per word and σ² is its variance [28].

Table 1 shows the interpretation of the scores of a text according to its level of readability.

To perform readability analysis of the included questionnaires, the instrument https://legible.es/ (accessed 10 February 2021) was used; this program is a free Python script (its license is General Public License 3) and it is a tool that can be used to check the readability of a text or a URL.

## 3. Results

Table 2 shows the questionnaires studied, as well as their main characteristics and number of questions.

Table 3 shows the readability results obtained for the questionnaires that evaluate symptoms related to different pelvic floor dysfunctions.

The most relevant data are shown in questionnaires that assess fecal incontinence, where 50% of questionnaires (Browning and Parks and St Mark’s Hospital) obtained scores below the legibility limit in all indices. If we used the readability mu index as a reference, the number of questionnaires that did not meet the minimum readability limit increased, i.e., 20% in urinary incontinence, 50% in fecal incontinence, 66.6% in sexual function, 100% in general pelvic floor, 25% in overactive bladder and 100% in benign prostatic hyperplasia.

The mean of the scores for each index and for each dysfunction can be found next to each questionnaire in Table 3. The highest mean scores were found in the urinary incontinence questionnaires (except for the readability mu, where the overactive bladder questionnaires obtained the highest mean scores), while the lowest was obtained for fecal incontinence (in the case of the readability mu index, general pelvic floor dysfunction questionnaires).

**Figure 1:**
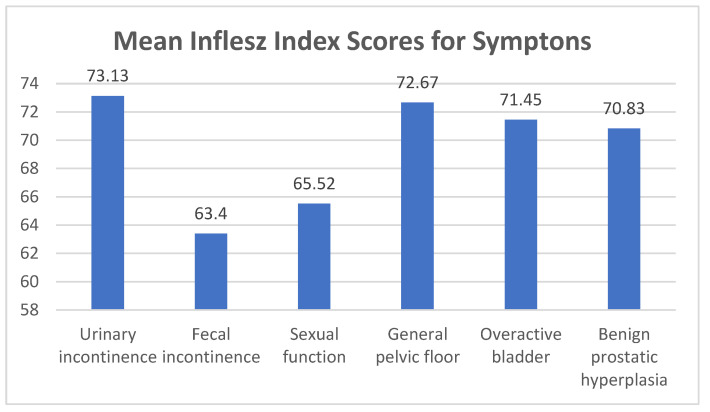
Mean of the scores according to the Inflesz index for questionnaires on symptoms.

Figure 1 reflects the mean of the readability scores according to the Inflesz index obtained for this group of questionnaires, divided according to the dysfunction to which they refer.

A higher and, therefore, better score in terms of readability was found for the questionnaires that evaluate symptoms related to urinary incontinence, while the lowest score was found for those aimed at evaluating symptoms related to fecal incontinence.

## 4. Discussion

All of the studies comparing the ICIQ-SF questionnaire for urinary incontinence to other questionnaires found adequate readability scores, except when compared with the UDI-6 and QUID questionnaires, where this study found an adequate readability score, in contrast to Alas et al. [8] and Gaines and Malik [5], who rated it as inadequate in terms of readability.

In the case of fecal incontinence questionnaires, the FISI questionnaire offered good readability scores across all studies.

Among the questionnaires that evaluate general disorders of the pelvic floor and sexual function, EPIQ was the only questionnaire with consistently good readability scores across all of the compared studies. Our readability results for the PFDI-20, PISQ-IR and PISQ-12 questionnaires contrast to those of Alas et al. [8] (though, in the case of the first two, if the readability mu index is taken as a reference, the results of all studies are aligned).

The IPSS questionnaire for benign prostatic hyperplasia produced normal results across all of the different tests.

Although better results were obtained for the readability of the questionnaires in Spanish, it is necessary to make certain observations in this regard. It is important that the information offered to patients during consultation or possible investigations, as well as that provided when they are asked to fill in a certain questionnaire, is optimal in terms of readability. This way, we can ensure that it is understood and responded to appropriately and, thus, can satisfactorily evaluate the symptoms related to the evaluated pelvic floor dysfunction. Among the other measures, it is necessary to determine which questionnaires have better readability indices and can best help with our evaluation. In this study, the scores obtained for most of the questionnaires analyzed (almost 90%) are considered to be adequate and consistent. Among the four readability indices used, there was a reliable general agreement for at least three, though there were discrepancies with the readability mu index. This could be explained by the different application formulas for each index. In the Fernández-Huerta, Szigriszt-Pazos and Inflesz indices, syllables, words by phrases and number of phrases are taken into account, while, for the readability mu index, the number of words, the mean of the letters per word and their variance are considered, which could yield different data from the other indices. When carrying out the analysis of the questionnaires, it is seen, therefore, that the use of the first three indices translates similar scores and the index mu more disparate ones than the previous ones. This is due to the differences in the parameters analyzed in the readability of a text. This is the reason why we consider the choice of a definite index adequate to analyze the comparisons between the different questionnaires and, due to its application in texts related to the health field, as well as the great precision in the study of Inés Barrios [26], the Inflesz index is considered to be the one that provides greater reliability in the analysis of results; therefore it appears to be the most reliable for future research that analyzes the legibility of texts in healthcare. Furthermore, according to the doctoral thesis of Inés Barrio, the perspicuity formula of Szigriszt-Pazos requires adaptation because its interpretation was carried out with an insufficient, non-representative, or random sample of texts. It is precisely this adaptation that the author made in her doctoral thesis work.

Theoretically, the lower the readability scores according to the indices used, the worse the comprehension on the part of the patients due to difficulty in reading. However, it would be an error to consider readability the only aspect that determines how well a text is understood, without taking into account other variables or factors, such as the language used and structure of the items, the presence or not of illustrative graphics, or the learning itself after reading and filling in the same questionnaire on more than one occasion, as well as the possible clarifications that an instructor could offer for a better answer. Indeed, a limitation of this study is that it does not consider these aspects. Potential ways to improve the readability of health questionnaires could be incorporating graphics or figures that facilitate the understanding of text, placing a limit of a maximum of 10 words per sentence, replacing medical terminology with its definition, as well as limiting medical jargon [29].

Another possible limitation of this study is that the questionnaires analyzed were not administered to subjects. Thus, a prospective line of research could be administering questionnaires that evaluate the same pelvic floor dysfunction in patients who are suspected or known to suffer from it and that analyze and compare the results of the different questionnaires.

## 5. Conclusions

Regarding questionnaires that evaluate symptoms associated with pelvic floor dysfunction, with the exception of those aimed at evaluating fecal incontinence (specifically the Browning and Parks and St. Mark’s Hospital questionnaires), the rest were found to have, at minimum, a normal score in terms of readability.

Apart from this, a possible re-evaluation of the questionnaires used in research and in practice for the identification of pelvic floor dysfunctions would be appropriate in order to facilitate the reading, understanding and collection of data by health professionals engaged in this area as much as possible.

This measurement should be taken into account when validating questionnaires to facilitate understanding and readability for patients with symptoms associated with pelvic floor musculature dysfunction.

## Figures and Tables

**Table 1:** Interpretation of the scores according to the readability index.

Lecturability Fernández Huerta	Level	Perspicuity Szigriszt-Pazos	Level	Perspicuity Inflesz	Level	Legibility µ	Level
90–100	Very easy	86–100	Very easy	80–100	Very easy	91–100	Very easy
80–90	Easy	76–85	Easy	65–80	Somewhat easy	81–90	Easy
70–80	Some easy	66–75	Somewhat easy	55–65	Normal	71–80	Somewhat easy
60–70	Normal	51–65	Normal	40–55	Somewhat difficult	61–70	Adequate
50–60	Somewhat difficult	36–50	Somewhat difficult	0–40	Very difficult	51–60	Somewhat difficult
30–50	Difficult	16–35	Difficult			31–50	Difficult
0–30	Very difficult	0–15	Very difficult			0–30	Very difficult

**Table 2:** Questionnaires analyzed, their items and their main characteristics.

Questionnaire	Dysfunction	Number of Questions	Characteristics
ICIQ-SF [9]	Urinary incontinence	3	Measures the frequency, quantity and affectation
UDI-6 [10]	Urinary incontinence	6	Grade of recommendation A; measures the degree of severity
3IQ [11]	Urinary incontinence	3	Classifies urge or stress incontinence in primary care settings
QUID [11]	Urinary incontinence	6	Has two subscales: stress and urgency
Severity test of Sandvik [12]	Urinary incontinence	2	Measures aspects of frequency and quantity
WEXNER [13]	Fecal incontinence	3	Collects data on the type of leak, use of dressings and lifestyle alteration
Browning y Parks [13]	Fecal incontinence	1	Divided into four degrees
FISI [13]	Fecal incontinence	1	A table is obtained with values between 0 and 61 points
St Mark’s Hospital [14]	Fecal incontinence	5	Evaluates use of astringent medication, diapers, or degree of urgency
FSFI [15]	Sexual function	19	Evaluates six domains: desire, arousal, lubrication, orgasm, satisfaction and pain
PISQ-IR [16]	Sexual function	12/21	12 items for sexually inactive women; 21 items for sexually active women
PISQ-12 [17]	Sexual function	12	Evaluates women with POP and/or urinary incontinence
OABSS [18]	Overactive bladder	7	Five items for urinary urgency and two for daily and nocturnal frequency
B-SAQ [19]	Overactive bladder	8	Divided into two scales that report discomfort and symptoms
OAB-V8 [20]	Overactive bladder	8	Measures the magnitude of affectation of the symptoms; a dichotomous item assesses the gender of the patient
OAB-V3 [20]	Overactive bladder	3	Neutral with respect to the gender of the patient
IPSS [21]	Benign prostatic hyperplasia	8	Seven items measure frequency and severity of symptoms and one item measures the general impact on quality of life
EPIQ [22]	General pelvic floor symptoms	22	Seven dimensions: Overactive bladder, stress urinary incontinence, quality of life, pelvic prolapse, incontinence, emptying pain and difficulty and defecatory dysfunction
PFDI-20 [23]	General pelvic floor symptoms	20	Divided into three scales: prolapse, colorectal–anal and urinary symptoms.

**Table 3:** Readability results for the questionnaires on symptoms.

Questionnaires	Fernández Huerta	Szigriszt-Pazos	Inflesz	Legibility µ
Normal readability limit	61	51	56	61
**Urinary incontinence**	77.68	73.13	73.13	68.62
ICIQ-SF	81.39	77.08	77.08	71.51
UDI-6	61.89	56.50	56.50	58.57 *
3 IQ	75.50	70.87	70.87	61.63
QUID	77.15	72.80	72.80	66.29
Severity test of Sandvik	92.47	88.41	88.41	85.11
**Fecal incontinence**	67.90	63.40	63.40	58.17
Wexner	99.92	95.96	95.96	76.24
Browning and Parks	44.12 *	38.98 *	38.98 *	47.34 *
FISI	73.49	69.43	69.43	63.36
St Mark´s Hospital	54.08 *	49.24 *	49.24 *	45.73 *
**Sexual Function**	70.03	65.52	65.52	59.64
FSFI	68.65	63.87	63.87	55.23 *
PISQ-IR	71.56	67.42	67.42	54.75 *
PISQ 12	69.87	65.26	65.26	68.94
**Overactive bladder**	76.07	71.45	71.45	72.72
B-SAQ	72.09	67.01	67.01	59.44 *
OAB-V8	74.45	69.82	69.82	71.53
OAB-V3	77.69	73.17	73.17	86.52
OABSS-S	80.05	75.79	75.79	73.39
**Benign prostatic hyperplasia**	75.30	70.83	70.83	55.94
IPSS	75.30	70.83	70.83	55.94 *
**General pelvic floor symptoms**	77.20	72.67	72.67	51.62
EPIQ	72.36	67.69	67.69	56.87 *
PFDI-20	82.04	77.65	77.65	46.36 *

* Scores below the readability limit for each index.

## Data Availability

Not applicable.
